# A Prophylactic Noninvasive Ventilation Reduces Complications Following Minimally Invasive Coronary Surgery

**DOI:** 10.3390/jcm14248834

**Published:** 2025-12-13

**Authors:** Janusz Konstanty-Kalandyk, Anna Kędziora, Dominika Batycka-Stachnik, Piotr Śliwiński, Przemysław Ptak, Dorota Sobczyk, Jacek Piątek

**Affiliations:** 1 Department of Cardiovascular Surgery and Transplantology, Saint John Paul II Hospital, Pradnicka 80, 31-202 Cracow, Poland; 2 Medical College, Jagiellonian University, sw Anny 12, 31-008 Cracow, Poland; 3 Department of Cardiac Surgery and Heart Transplantation, Wroclaw Medical University, ul. Borowska 213, 50-556 Wroclaw, Poland

**Keywords:** noninvasive ventilation, postoperative pulmonary complications, minimally invasive direct coronary artery bypass grafting

## Abstract

**Objective:** Postoperative pulmonary complications (PPCs) remain a significant source of morbidity and mortality in patients undergoing minimally invasive cardiothoracic procedures. Noninvasive ventilation (NIV) is frequently employed as adjunctive therapy to manage respiratory insufficiency. This study evaluated the implementation of prophylactic NIV immediately following extubation after minimally invasive direct coronary artery bypass (MIDCAB) surgery. **Methods:** A total of 454 consecutive patients undergoing MIDCAB were included. In total, 139 patients received prophylactic NIV (P-NIV)—postoperative management, 315 patients formed a historical control group treated according to the previous standard of care. Clinical outcomes assessed postoperative pulmonary complications, in-hospital mortality, and one-year survival. **Results:** The incidence of PPCs was significantly lower in the P-NIV group compared with the control cohort (6.5% vs. 14.9%; *p* = 0.012). Unadjusted analyses demonstrated a significant reduction in the odds of PPCs with P-NIV (odds ratio [OR], 0.39; 95% confidence interval [CI], 0.17–0.85). Using inverse probability of treatment weighting, prophylactic NIV was associated with an absolute reduction of 8.0 percentage points in PPC risk across the entire cohort (average treatment effect [ATE], −0.080; 95% CI, −0.136 to −0.024; z = −2.80; *p* = 0.005). Kaplan–Meier analysis demonstrated significantly improved one-year survival in the P-NIV group (log-rank *p* = 0.047). **Conclusions:** The implementation of prophylactic NIV following MIDCAB was associated with a greater than 50% reduction in the odds of PPCs in both unadjusted and adjusted analyses and improved one-year survival. These results support the adoption of routine prophylactic NIV in the postoperative management of patients undergoing minimally invasive coronary surgery.

## 1. Introduction

Postoperative pulmonary complications (PPCs) encompass a diverse spectrum of respiratory conditions, ranging from minor to severe. The overall incidence of PPCs in patients undergoing cardiothoracic surgery is notably high, estimated between 30% and 50% [1,2]. Findings from the VENICE International Cohort Study, involving 707 patients across 43 cardiac surgery centers in nine countries, corroborated the upper range of this estimate—reporting that 55% of patients experienced at least one PPC following cardiac surgery [3]. This high incidence is attributed mainly to the inherent nature of cardiac surgery, which involves direct intrathoracic manipulation. Postoperative structural and functional alterations of the chest wall further contribute to impaired lung expansion and exacerbate pulmonary atelectasis. In fact, among the most frequently reported PPCs are atelectasis, with an incidence ranging from 16.6% to 88%, followed by pneumonia, occurring in 2.1% to 21.6% of cases. Moreover, the clinical implications of PCCs for patient outcomes hold paramount importance. In this context, PCCs have emerged as a significant contributor to postoperative morbidity and mortality, particularly in the setting of cardiac surgery. [4].

Contemporary cardiac surgery prioritizes reducing perioperative trauma, fostering a rise in minimally invasive approaches. One such technique, minimally invasive direct coronary artery bypass grafting (MIDCAB), is gaining traction due to its ability to circumvent sternotomy, aortic manipulation, and cardiopulmonary bypass (CPB), while achieving comparable graft patency rates to conventional coronary surgery [5]. In Poland, an average of over 500 MIDCAB procedures have been performed annually in recent years, accounting for more than 5% of total coronary operations, a number expected to rise with increasing interest in hybrid revascularization strategies. However, the MIDCAB approach necessitates single-lung ventilation for a substantial portion of the procedure. This ventilation technique may be associated with an elevated risk of PPCs, attributable to incomplete postoperative lung re-expansion, particularly in cases when postoperative pulmonary rehabilitation is suboptimal.

Numerous studies have highlighted the significance of noninvasive ventilation (NIV) as an adjunctive therapy for treating postoperative pulmonary complications and respiratory insufficiency [6]. By providing positive airway pressure during both inspiration and expiration, NIV facilitates alveolar recruitment, prevents alveolar collapse, mitigates ventilation-perfusion mismatch, and optimizes cardiac function by reducing left ventricular preload and afterload [7]. Despite its potential benefits, the routine prophylactic use of NIV following MIDCAB procedures remains largely unexplored and warrants further investigation to elucidate its role in improving postoperative pulmonary outcomes. This study aims to address the existing gap in evidence by analyzing clinical outcomes following the implementation of prophylactic NIV as an immediate post-extubation strategy in a high-volume MIDCAB center, to enhance postoperative pulmonary recovery and reduce the incidence of pulmonary complications, in comparison to the previous standard of care.

## 2. Materials and Methods

A total of 454 consecutive patients who underwent MIDCAB at the Department of Cardiovascular Surgery and Transplantology, Saint John Paul II Hospital in Krakow, between 2019 and 2024 were included in this analysis. All patients underwent a MIDCAB procedure with the left internal mammary artery (LIMA) to the left anterior descending (LAD) artery, utilizing a direct vision LIMA harvest. All procedures were performed on a beating heart, using a tissue stabilizer, as previously reported [8]. Intraoperative single-lung ventilation was delivered in all cases via intubation with a standard double-lumen tube and restricting airflow to the right lung throughout the majority of the procedure. Reintubation with a single-lumen tube was performed at the conclusion of each procedure, with lung expansion visualized directly before chest closure. Patients were subsequently extubated in the ICU once deemed appropriate, with a target of total ventilation time not exceeding 6 h, per Enhanced Recovery After Cardiac Surgery (ERAS^®^ Cardiac) recommendations.

Since April 2022, an institutional protocol for prophylactic NIV has been implemented in all patients undergoing MIDCAB to systematically reduce the incidence of PCCs in this group. Prior to the implementation of this protocol, between 2019 and April 2022, the former standard of care guided postoperative pulmonary rehabilitation, and NIV was utilized solely as an adjunctive therapy in patients presenting with postoperative respiratory complications, including atelectasis, reduced oxygen saturation, or other significant ventilatory impairments.

### 2.1. Prophylactic NIV Protocol

The protocol consisted of five two-hour sessions of NIV using Bilevel Positive Airway Pressure (BIPAP), implemented to enhance postoperative pulmonary recovery. This intervention was introduced in addition to the prior standard of care, which had relied solely on respiratory exercises.

The NIV intervention commenced 20–30 min post-extubation, following an initial 30-min session of individualized respiratory therapy conducted by a physiotherapist, which primarily focused on breathing exercises and secretion clearance. Upon completion of this preparatory session, patients received their first NIV application. The remaining four sessions were administered over the first and second postoperative days, with a frequency of two sessions per day.

NIV therapy was delivered in ST mode, with the following initial parameters:

- EPAP at 5–6 cm H_2_O,
- IPAP titrated within the range of 10–15 cm H_2_O to achieve a target tidal volume (TV) of 6–8 mL/kg ideal body weight,
- FiO_2_ adjusted within a range of 35–50% to optimize oxygenation.

Further adjustments were made as necessary, guided by clinical parameters such as arterial blood gas analysis, chest radiographic findings, and the presence of a pre-existing diagnosis of sleep apnea, to ensure optimal respiratory rehabilitation.

### 2.2. Former Pulmonary Rehabilitation Protocol

The prior standard of care for postoperative pulmonary rehabilitation was predominantly based on a multimodal approach incorporating breathing exercises, bubble positive expiratory pressure (PEP), incentive spirometry, cough enhancement techniques, secretion clearance strategies, and early mobilization, all aimed at facilitating effective respiratory recovery. These respiratory exercises were conducted with a frequency comparable to that of the post-protocol implementation group.

The primary outcome was the incidence of any PPC during the index hospitalization. To assess this, all patients’ medical records—including radiographic findings—were systematically reviewed. PPCs were defined according to the criteria established by the European Joint Task Force [9], and included the following:

- respiratory infection: administration of antibiotics for suspected infection, accompanied by at least one of the following—new or altered sputum, radiographic evidence of lung opacities, fever, or leukocytosis (WBC > 12 × 10^9^/L)
- pleural effusion: radiographic signs such as blunting of the costophrenic angle, obscured hemidiaphragm silhouette, anatomical displacement, or hazy opacity in the supine position
- atelectasis: radiographic signs, such as lung opacification, with associated shift of the mediastinum, hilum, or diaphragm, along with compensatory hyperinflation
- pneumothorax: radiographic evidence of air in the pleural space with absent vascular markings
- pleural puncture: a therapeutic intervention performed for pleural effusion

The secondary outcome was all-cause mortality within one year following surgery, determined using verified data from the Polish National Registry of Cardiac Surgical Procedures (KROK).

### 2.3. Statistical Analysis

Categorical variables are reported as counts (percentages) and compared by the χ^2^ test. Continuous variables are expressed as mean ± SD or median (IQR) and compared by Student’s *t*-test or Mann–Whitney U test, as appropriate. The unadjusted odds ratio (OR) was calculated to estimate the association between prophylactic noninvasive ventilation (P-NIV) and the incidence of postoperative pulmonary complications (PPCs). To control for potential confounding, Mantel-Haenszel odds ratios were stratified by age category, sex, obesity, chronic lung disease, preoperative mobility status, and re-exploration for bleeding. A time-trend analysis was performed using univariate logistic regression to assess the effect of the year of surgery on the incidence of postoperative pulmonary complications. A multivariable logistic regression model, including age, sex, chronic lung disease, poor mobility, re-exploration for bleeding, and ICU length of stay, was then fitted to adjust for covariates identified in the univariate analysis and clinically relevant covariates. Inverse probability of treatment weighting (IPTW) was applied to estimate the average treatment effect (ATE) alongside the average treatment effect on the treated (ATET) of prophylactic noninvasive ventilation (P-NIV), with propensity scores derived from a multivariable logistic regression model in which receipt of P-NIV was the dependent variable and predefined covariates as the predictors—sex, chronic lung disease, poor mobility, heart failure, diabetes, re-exploration for bleeding, and postoperative ventilation time. Trimming the IPTW weights was performed in two steps. First, a trimmed weight variable was created by excluding all weights above the 95th percentile. Second, covariate balance in the trimmed sample was assessed using standardized mean differences of less than 0.1. One-year survival was analyzed by the Kaplan–Meier method, with group comparisons via the log-rank test. Statistical analysis was performed using Stata 18.5 (StataCorp LLC., College Station, TX, USA). All tests were two-tailed, with *p* < 0.05 denoting statistical significance.

## 3. Results

The prophylactic NIV protocol was implemented in April 2022, and from that point forward, 139 patients treated under this protocol (prophylactic NIV–P-NIV group) were compared with 315 patients who had received the prior standard of care for postoperative pulmonary rehabilitation (historical cohort). Overall, the baseline characteristics were well balanced between the groups (Table 1), and a similar degree of comparability was observed in the peri-procedural data (Table 2). In both cohorts, the majority of patients were male, with mean ages of 68 and 67 years, respectively. The procedures were predominantly performed electively, and approximately one-third of patients in each group underwent MIDCAB as part of a hybrid revascularization approach. Patients in both groups were extubated within the early postoperative hours, with median extubation times of 5 and 7 h, respectively, and prolonged mechanical ventilation was infrequently required.

**Figure 1:**
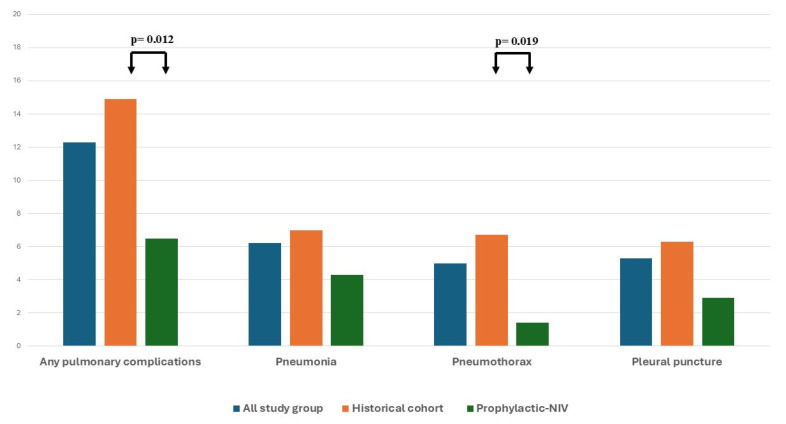
Postoperative pulmonary complications.

Postoperative pulmonary complications were observed in 14.9% of the historical control cohort versus 6.5% of the prophylactic NIV cohort (*p* = 0.012), with the most significant intergroup divergence noted in the incidence of postoperative pneumothorax (Figure 1). Unadjusted analysis demonstrated a significant reduction in the odds of PPCs with prophylactic NIV (OR, 0.39; 95% CI, 0.17–0.85). Stratified Mantel–Haenszel estimates confirmed a consistent protective effect across all examined subgroups—age and weight categories, sex, chronic lung disease, mobility status, and need for re-exploration for bleeding—with odds ratios clustered around 0.4, underscoring the uniform benefit of P-NIV (Table 3).

After controlling for key confounders—age, sex, chronic lung disease, poor mobility, postoperative bleeding re-exploration, and ICU length of stay (selected based on univariate significance or clinical relevance)—prophylactic NIV remained associated with a consistent, significant reduction in postoperative pulmonary complications (adjusted OR 0.44, 95% CI 0.20–0.98). Notably, re-exploration for bleeding was associated with a markedly elevated risk of PPCs, underscoring that meticulous hemostasis and avoidance of repeat chest opening are critical to effective PCC prophylaxis (Table 4). Importantly, although the protocol change was implemented over successive years, the year of surgery itself did not predict complication rates in a univariate analysis (OR 0.93, 95% CI 0.79–1.07), underscoring the robustness of the observed effect independent of temporal trends.

**Figure 2:**
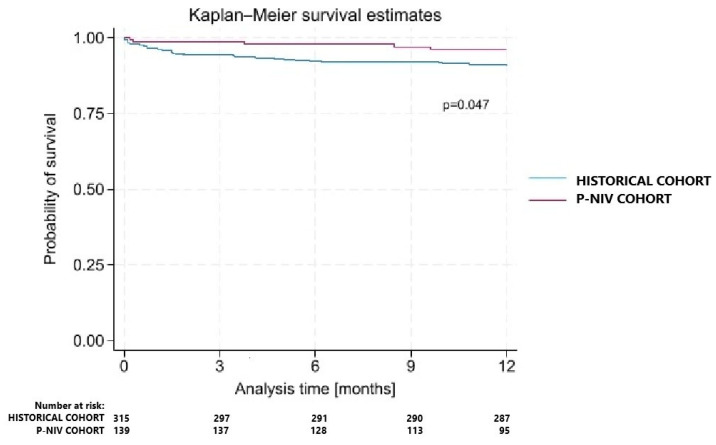
1-year follow-up results.

Using IPTW, the average treatment effect of P-NIV was an 8.0-percentage-point reduction in PPC risk in the overall cohort (ATE, −0.080; 95% CI, −0.136 to −0.024; z = −2.80; *p* = 0.005) and a 6.7-percentage-point reduction among treated patients (ATET, −0.067; 95% CI, −0.124 to −0.010; z = −2.30; *p* = 0.021). Although early postoperative mortality did not differ significantly, Kaplan–Meier analysis demonstrated superior one-year survival in the P-NIV group compared with the historical cohort (log-rank *p* = 0.047; Figure 2), suggesting that routine prophylactic NIV may confer durable long-term benefits after MIDCAB.

## 4. Discussion

Despite the alarmingly high incidence of pulmonary complications following cardiac surgery, prophylactic strategies remain insufficiently emphasized and underutilized in both clinical practice and the literature [1,2]. Postoperative pulmonary complications represent a wide range of clinical scenarios, ranging from commonly reported atelectasis and hypoxemia to overt acute respiratory distress syndrome (ARDS). Although atelectasis and small pneumothorax are exceedingly common after cardiac surgery, they predispose patients to postoperative pneumonia and delay recovery by exacerbating pulmonary distress, impeding rehabilitation, and limiting physical capacity [10]. Our historical cohort’s complication rate aligns with published experience in conventional cardiac surgery (20%) [2], at around 15%, and compares favorably to minimally invasive valve surgery cohorts lacking a routine prophylactic NIV approach [1], where the PCC rate was higher, at 19%. Additionally, a notably lower incidence was observed in the P-NIV group, with postoperative pulmonary complications occurring in approximately 6.5% of patients. Beyond the frequency alone, its consequences on patient outcomes are substantial. PCCs have been associated with increased mortality after noncardiac procedures and are known to increase length of stay and overall healthcare costs [2,11].

NIV is now an established first-line therapy for acute hypoxemic respiratory failure, delivering positive airway pressure to recruit collapsed alveoli, optimize ventilation–perfusion matching, and unload fatigued respiratory muscles. Its efficacy has also been demonstrated in acute heart failure—particularly cardiogenic pulmonary oedema with concomitant atelectasis—by improving alveolar recruitment, reducing both preload and afterload, and alleviating inspiratory muscle workload [7]. The concept of prophylactic noninvasive respiratory support to enhance postoperative pulmonary rehabilitation is also by no means novel. A recent frequentist network meta-analysis of 16 randomized trials compared immediate postoperative use of NIV, CPAP, HFNC, or standard care after cardiac surgery and found that NIV provided the most significant reduction in pulmonary complications [12]. Nevertheless, the study identified a low overall certainty of evidence and concluded that further high-quality research is required to delineate the comparative benefits of each noninvasive ventilatory support modality. Moreover, conventional cardiac surgery—performed via median sternotomy with cardiopulmonary bypass—induces systemic inflammation, hemodilution, and fluid overload, and necessitates intraprocedural cessation of ventilation, thereby profoundly altering postoperative respiratory physiology and limiting the direct applicability of its findings to the minimally invasive direct coronary artery bypass (MIDCAB) setting. Such a focused investigation is of paramount importance, as it uniquely addresses the MIDCAB population, a procedure performed increasingly worldwide and serving as a key component of the burgeoning hybrid revascularization paradigm, thereby filling a critical gap in the current scientific evidence.

Although MIDCAB aims to minimize surgical trauma and hasten functional recovery, PCCs remain a clinically important outcome in this population. Yet, PCC rates are seldom emphasized in MIDCAB studies, where the primary focus has been on shortened hospital stay, reduced transfusion requirements, and long-term cardiovascular endpoints such as graft patency and myocardial infarction [13]. At our institution, prophylactic NIV has been introduced routinely after MIDCAB surgeries over the past two years. Implementation of the prophylactic NIV protocol with BIPAP settings was associated with an unadjusted odds ratio of 0.39 for postoperative pulmonary complications, reflecting a decrease in overall complication rates from 14.9% to 6.5% (*p* = 0.012). The most pronounced decrease was seen in postoperative pneumothorax, which fell from over 6% to under 2%, while pneumonia and pleural puncture incidences were similarly reduced by approximately 50%. Notably, the rate of adjunctive respiratory physiotherapy remained stable between the pre- and post-prophylactic NIV periods, and the year of surgery exerted no significant effect, thereby underscoring prophylactic NIV as the principal determinant of the observed improvement. These findings align with those of Zhou et al., who reported that postoperative prophylactic NIV significantly reduces the incidence of pulmonary complications in contemporary cardiac surgery patients [12]. Similar benefits of prophylactic NIV have also been previously debated in terms of invasive thoracic procedures, which access mimics a MIDCAB setting, with a small thoracotomy and single-lung ventilation required throughout most of the procedure. In an extensive analysis of nearly 2000 patients that assessed the effect of prophylactic NIV after invasive thoracic procedures, including cardiac surgery, the authors concluded that NIV reduces postoperative pulmonary complications but does not affect hospital mortality [14]. Additionally, it has been demonstrated that the use of NIV does not increase the risk of adverse events compared to usual respiratory care. A further beneficial impact, presenting beyond only in-hospital outcomes, was presented by Pettenuzzo et al., who concluded that NIV reduced both in-hospital and long-term mortality in patients with post-extubation respiratory failure compared to conventional oxygen therapy [15].

Nevertheless, the incidence of PCC reflects a complex interplay of patient- and procedure-related factors, many of which are only partially modifiable. Baseline comorbidities—particularly advanced age, impaired mobility, and chronic obstructive pulmonary disease —substantially increase PCC risk by eroding respiratory reserve and hampering effective deep breathing and cough [16]. However, Mantel–Haenszel stratified analyses conducted in this study showed that a prophylactic NIV strategy significantly reduced PCC rates across all baseline risk-factor strata, including elder age, poor mobility, and diagnosis of chronic lung disease, with pooled ORs clustering consistently around 0.4 in each subgroup. Another significant contributor to PCC risk was re-exploration for bleeding, which emerged as the strongest predictor of PCC, conferring nearly a fourfold increase in risk. Notwithstanding this elevated risk, risk-adjusted analyses demonstrated that prophylactic NIV retained its protective effect despite the necessity for re-exploration. Although the small sample size of this subgroup constrained further analytical assessments, it is broadly acknowledged that those at highest risk for complications commonly derive the greatest benefit from a structured prophylactic strategy.

Finally, as perioperative mortality following cardiac surgery has declined markedly over the past decades, demonstrating an immediate survival benefit for prophylactic interventions has become increasingly challenging. Consequently, attention has shifted toward one-year outcomes as a more sensitive barometer of durable health gains. Postoperative pulmonary complications have been independently linked to elevated perioperative mortality after cardiac surgery. Moreover, PCCs are associated with increased mortality in noncardiac surgical cohorts [11]. Although short-term mortality rates remained low and statistically unchanged in our study, routine postoperative NIV in noncardiac settings corresponded with a downward trend in hospital mortality [12] and a significant improvement in one-year survival—findings that mirror the long-term mortality benefits reported in Pettenuzzo et al.’s meta-analysis of prophylactic NIV [15].

To our knowledge, the presented study is the first to demonstrate that prophylactic use of NIV has a beneficial effect on 1-year mortality in patients after minimally invasive coronary artery surgery (MIDCAB).

Overall, this study presents a detailed, successfully implemented prophylactic NIV protocol that yielded a substantial reduction in PCC rates following MIDCAB surgery. However, it is not without limitations. The single-center design may constrain external validity; nonetheless, by originating from a high-volume MIDCAB center, the study benefits from rigorous procedural consistency and minimized variability in both surgical technique and perioperative management. Undoubtedly, the natural progression of surgical expertise and the increasing experience of the multidisciplinary team in postoperative care may have contributed to improved patient outcomes, representing a potential confounding factor. However, multiple risk-adjustment estimations were performed to ensure the robustness of clinical findings, including the potential impact of year-based time trends. Therefore, despite these limitations, this study is the first to systematically investigate the potential clinical benefits of a postoperative prophylactic NIV strategy in minimizing the risk of pulmonary complications following MIDCAB surgery. Consequently, the study provides a strong foundation for future randomized controlled trials, enabling further validation of its findings in broader, multi-center settings.

### Study Limitations

This research is not a randomized clinical trial; it is a retrospective study that compares two historical cohorts with risk of temporal bias and inherent improvement of care over time. Although the year of intervention did not impact the incidence of PPC (OR 0.93), this does not completely eliminate the risk of confounding.

## 5. Conclusions

In conclusion, prophylactic NIV was associated with a more than 50% reduction in the odds of postoperative pulmonary complications after MIDCAB—an effect that remained consistent across patient subgroups and robust to multivariable adjustment. Although early postoperative mortality did not differ, one-year survival was significantly higher in the P-NIV cohort, suggesting a durable long-term benefit. These findings support the incorporation of prophylactic NIV into enhanced-recovery pathways for cardiac surgery and underscore the need for prospective studies to optimize its timing and application.

## Figures and Tables

**Table 1:** Baseline characteristics.

	P-NIV n = 139	Historical Cohort n = 315	*p*
Age, years	68 (62–73)	67 (61–73)	0.637
Male sex, n (%)	110 (79.1)	244 (77.5)	0.691
Euroscore II	1.46 (1.05–2)	1.26 (0.87–1.75)	0.003
Hybrid coronary procedure, n (%)	46 (33.3)	100 (31.7)	0.739
BMI, kg/m^2^	27.64 (25.42–29.76)	27.68 (25.01–30.48)	0.777
Hypertension, n (%)	121 (87.1)	296 (94)	0.013
Heart failure, n (%)	36 (25.9)	57 (18.1)	0.058
Atrial fibrillation, n (%)	29 (20.9)	55 (17.5)	0.389
Peripheral artery disease, n (%)	29 (20.9)	67 (21.3)	0.922
Diabetes, n (%)	46 (33.1)	122 (38.7)	0.252
Diabetes on insulin, n (%)	10 (7.2)	32 (10.2)	0.315
Chronic lung disease, n (%)	5 (3.6)	23 (7.3)	0.130
Contraindications to median sternotomy, n (%)	44 (62.9)	158 (56.4)	0.330

Values are presented as n (%) for categorical variables and as median (IQR) for quantitative variables. P-NIV—prophylactic NIV.

**Table 2:** Perioperative data.

	P-NIV n = 139	Historical Cohort n = 315	*p*
Mechanical ventilation time, hours	5 (4–7)	7 (5–8)	<0.001
Prolonged mechanical ventilation *, n (%)	1 (0.7)	2 (0.6)	0.918
Re-exploration for bleeding, n (%)	6 (4.3)	24 (7.6)	0.192
Renal complications, n (%)	7 (5)	12 (3.8)	0.548
In-hospital mortality, n (%)	2 (1.4)	12 (3.8)	0.178
Cardiac death	0	6 (1.9)	0.101
Noncardiac death	2 (1.4)	6 (1.9)	0.728

Values are presented as n (%) for categorical variables and as median (IQR) for quantitative variables. P-NIV—prophylactic NIV. * Prolonged mechanical ventilation is defined as >24 h.

**Table 3:** Crude and stratified odds ratios for Postoperative Pulmonary Complications (P-NIV Group vs. Historical Standard of Care).

Crude analysis	0.39 (0.17–0.85)
Stratified analysis *	
Age < 65 vs. ≥65 years	0.38 (0.18–0.81)
Sex	0.40 (0.19–0.84)
Chronic lung disease	0.41 (0.19–0.88)
Poor mobility	0.40 (0.19–0.85)
Re-exploration for bleeding	0.43 (0.20–0.91)

Odds ratios (OR) with 95% CI are presented. * Mantel-Haenszel Estimates.

**Table 4:** Multivariate adjusted analysis for Postoperative Pulmonary Complications.

P-NIV	0.44 (0.20–0.98)
Age	1.01 (0.97–1.04)
Female	0.53 (0.22–1.31)
Chronic lung disease	2.05 (0.71–5.96)
Poor mobility	1.15 (0.32–4.11)
Re-exploration for bleeding	3.24 (1.24–8.47)
ICU lenght of stay	1.33 (1.15–1.53)

Odds ratios (OR) with 95% CI are presented. Multivariate logistic regression model—likelihood-ratio χ^2^ *p* < 0.001; AUC = 0.75.

## Data Availability

The data underlying this article will be shared on reasonable request to the corresponding author.

## Abbreviations

The following abbreviations are used in this manuscript:

PPCs	postoperative pulmonary complications
MIDCAB	minimally invasive direct coronary artery bypass grafting
CPB	cardiopulmonary bypass
NIV	noninvasive ventilation
LIMA	left internal mammary artery
BIPAP	bilevel positive airway pressure
PEP	positive expiratory pressure
